# Chicago College of Pharmacy

**Published:** 1867-04

**Authors:** J. W. Mill

**Affiliations:** Secretary


### CHICAGO COLLEGE OF PHARMACY.

A very great interest is just now being manifested among the druggists of the City with reference to this still living, but almost forgotten, institution. The College has recently been the recipient of a costly and extensive collection of chemical specimens, the gift of Messrs. POWERS & WEIGHTMAN, the well-known manufacturing chemists of Philadelphia.

The inspection of these specimens was made the occasion of an informal meeting last Wednesday afternoon. From the remarks made by the President, Mr. E. H. SARGENT, it appears that, besides chairs, cases for specimens, etc., the College is in possession also of quite a variety of specimens of materia medica, to which additions have been promised as soon as the College is in proper condition to receive them. These, with the valuable collection above referred to, constitute an ample basis upon which to build up a cabinet that will be an honor to the name and fame of our City.

It is proposed also, to organize a library, to contain the best works on pharmacy and the collateral sciences. It is not considered best to attempt a course of lectures at present, or until the College has become strong enough to make them successful beyond a doubt. The surplus funds will be used in procuring books, specimens, etc. At the conclusion of the President's remarks, Messrs BUCK, SWEET, and EHRMAN were appointed a committee to nominate officers. The following were reported and elected:—

*President*—Mr. E. H. SARGENT.

*Vice-Presidents*—Messrs GEORGE BUCK and W. H. MULLER.

*Secretary*—Mr. JAMES W. MILL.

*Treasurer*—Mr. J. P. SHARP.

*Trustees*—ALBERT E. EBERT, HENRY SWEET, JOHN W. EHRMAN, WILLIAM REINBOLD, and EMIL DREIER.

Messrs WHITFIELD, EHRMAN, and BLOCKI were then appointed a special committee to secure permanent rooms for the meetings of the College. At this stage of the proceedings, a member of the profession from Leavenworth, Kansas, was introduced to the meeting by Mr. EBERT, and, on motion, unanimously elected an associate member.

The large attendance, the enthusiastic and hopeful spirit manifested at this meeting, augur well for the future success of the College. The other large cities of the Union—Philadelphia, New York, Boston, Baltimore, Cincinnati, and St. Louis—have their Colleges of Pharmacy. Why not Chicago? Let the druggists of Chicago have regard to their reputation for intelligence and enterprise, and see to it that the impetus thus given to their College does not fail, from any supineness or indifference on their part, to carry it forward to a high position of usefulness and honor. It is to be hoped that not only the druggists of the City, but many, also, throughout the Northwest, will feel sufficient interest to identify themselves with the College, either as active or associate members. Copies of the constitution and by-laws can be had on application to the Secretary, JAMES W. MILL, 119 West Adams Street.

---

The regular monthly meeting of the Chicago College of Pharmacy was held on the afternoon of March 4th, 1867, in Room No. 20, Rice's Building, the President, E. H. SARGENT, in the chair. A large attendance of members present.

The minutes of the previous evening were read and approved.

The Committee on Renting Rooms reported that they had secured a desirable suite of rooms in Rice's Block, and that the same would be suitably furnished as soon as possible.

On motion, a committee was appointed to prepare a design for certificates of membership to be issued to each member of the College, as provided for in the by-laws.

On motion, standing committees were appointed on the progress of Pharmacy and the revision of Pharmacopœia; and, also, a temporary committee on the solicitation of specimens for the College.

The Secretary was on motion instructed to notify the different organizations throughout the Country of the reörganization of the Chicago College of Pharmacy.

Several contributions to the library were reported by Mr.

A. E. EBERT, and a vote of thanks was tendered to him for his donations.

M. EBERT read a paper on the preparation of Bibromide of Mercury, and on a substitute for Liebig's food for infants, made from wheat bran. The subject was fully discussed by the members present.

The meeting then adjourned to meet on Monday, March 18th, 1867.

J. W. MILL, *Secretary.*

Druggists throughout the North-west are earnestly invited to become members, and to contribute to the cabinet and library of the College.

